# IL-1β, But Not Programed Death-1 and Programed Death Ligand Pathway, Is Critical for the Human Th17 Response to *Mycobacterium tuberculosis*

**DOI:** 10.3389/fimmu.2016.00465

**Published:** 2016-11-04

**Authors:** Emmanuel Stephen-Victor, Varun Kumar Sharma, Mrinmoy Das, Anupama Karnam, Chaitrali Saha, Maxime Lecerf, Caroline Galeotti, Srinivas V. Kaveri, Jagadeesh Bayry

**Affiliations:** ^1^Institut National de la Santé et de la Recherche Médicale Unité 1138, Paris, France; ^2^UMR S 1138, Sorbonne Universités, UPMC Univ Paris, Paris, France; ^3^Centre de Recherche des Cordeliers, Equipe – Immunopathology and Therapeutic Immunointervention, Paris, France; ^4^UMR S 1138, Université Paris Descartes, Sorbonne Paris Cité, Paris, France

**Keywords:** PD-L1, PD-1, *Mycobacterium tuberculosis*, dendritic cells, monocytes, Th17, IL-23, IL-1β

## Abstract

The programed death-1 (PD-1)–programed death ligand-1 (PD-L1) and PD-L2 co-inhibitory pathway has been implicated in the evasion strategies of *Mycobacterium tuberculosis*. Specifically, *M. tuberculosis*-induced PD-L1 orchestrates expansion of regulatory T cells and suppression of Th1 response. However, the role of PD pathway in regulating Th17 response to *M. tuberculosis* has not been investigated. In the present report, we demonstrate that *M. tuberculosis* and *M. tuberculosis*-derived antigen fractions have differential abilities to mediate human monocyte- and dendritic cell (DC)-mediated Th17 response and were independent of expression of PD-L1 or PD-L2 on aforementioned antigen-presenting cells. Importantly, we observed that blockade of PD-L1 or PD-1 did not significantly modify either the frequencies of Th17 cells or the production of IL-17 from CD4^+^ T cells though IFN-γ response was significantly enhanced. On the contrary, IL-1β from monocytes and DCs were critical for the Th17 response to *M. tuberculosis*. Together, our results indicate that IL-1β, but not members of the programed death pathway, is critical for human Th17 response to *M. tuberculosis*.

## Introduction

Tuberculosis caused by *Mycobacterium tuberculosis* still remains a global threat with an estimated 1.5 million deaths annually. Cellular immunity plays a critical role in mediating the protection against tuberculosis. Indeed, IFN-γ-producing CD4^+^ T helper type 1 cells are critical for the control of *M. tuberculosis* in humans and murine models (1–4). Thus, general paradigm for tuberculosis vaccines has largely focused on enhancing the Th1/IFN-γ response (5, 6). However, despite enhancing IFN-γ response, the recombinant MVA85A vaccine failed to protect infants from tuberculosis (7). Therefore, it is pertinent to decipher the role played by other CD4^+^ T cell subsets and their cytokines in mediating immunity against *M. tuberculosis*.

Th17 cells that express transcription factor RORC and secrete archetype cytokine IL-17A represent a distinct subset of CD4^+^ T cells. Th17 cells also produce IL-17F, IL-21, IL-22, GM-CSF, and IL-26 and mediate pro-inflammatory responses (8–11). Th17 cells are associated with the pathogenesis of several autoimmune and inflammatory diseases (12–14). Evolutionarily, Th17 response is conserved to mediate protection at mucosal surfaces and against extracellular pathogens (10, 15). Recent reports have also indicated that Th17 response may play a crucial role in mediating protection against intracellular pathogens, such as *Francisella tularensis* and *Chlamydia muridarum* (16–18). These data thus indicate the diverse role of Th17 cells in various physiopathologies.

*Mycobacterium tuberculosis* employs a plethora of mechanisms to suppress both innate and adaptive immune responses. The role of Th17 response to *M. tuberculosis* is largely pursued in mice, and it remains highly controversial (19–25). Recent reports in tuberculosis patients indicate that active disease and its severity are associated with low Th17 response (26, 27). Of note, anti-tuberculosis therapy is associated with enhanced Th17 response, suggesting that *M. tuberculosis* suppresses Th17 response as one of the immune evasion mechanisms (28).

Programed death-1 (PD-1)–programed death ligand-1 (PD-L1)/PD-L2 pathway occupies a unique place in the immune evasion strategies employed by *M. tuberculosis*. Recent data highlight the role of PD-1–PD-L1/PD-L2 axis in modulating regulatory T cell (Treg) and Th1 response to *M. tuberculosis* (29–33). Whether this pathway also regulates Th17 response to *M. tuberculosis* is not known. Therefore, in the present study, we have evaluated the role of PD pathway members (PD-L1, PD-L2, and PD-1) in mediating human monocyte- and dendritic cell (DC)-mediated Th17 response to *M. tuberculosis*.

Several reports have shown that DCs promote Th17 responses to either *M. tuberculosis* or its antigens (34–37). We found that monocytes and DCs have differential capacity to promote Th17 response to *M. tuberculosis* and *M. tuberculosis*-derived antigens. Notably, a prominent IL-17 response was mediated by DCs in comparison to monocytes. Although both monocytes and DCs did not express PD-L2, PD-L1 was significantly enhanced upon stimulation with *M. tuberculosis*. Similarly, *M. tuberculosis* stimulation of monocyte/DC–CD4^+^ cocultures also lead to significant increase in the frequency of PD-1^+^CD4^+^ T cells. Importantly, blocking PD-L1 or PD-1 neither significantly altered the frequencies of Th17 cells nor augmented IL-17 secretion from CD4^+^ T cells. Analysis of key Th17-polarizing cytokines indicated that the production of IL-1β was crucial in the establishment of Th17 response to *M. tuberculosis*. These results thus reveal that the outcome of Th17 response to *M. tuberculosis* is dictated by the capacity of human innate cells to secrete key Th17-polarizing cytokine (IL-1β) and not expression of members of the PD pathway.

## Materials and Methods

### Antibodies

FITC-conjugated mAbs to CD86 [clone 2331 (FUN-1)], CD274 (clone MIH1), PE-conjugated mAbs to pSTAT3 (clone 4/P-STAT3), CD80 (clone L307.4), PD-L2 (clone 2D3/B7-H2), antigen-presenting cell (APC)-conjugated mAbs to HLA-DR (clone G46-6), PD-1 (clone MIH4), Alexa 700-conjugated mAb to CD4 (clone RPA-T4), and BV421-conjugated mAb to CD4 were from BD Biosciences (Le Pont de Claix, France). PE-conjugated mAbs to IL-17A (clone eBio64CAP17), human–mouse RORγt (AFKJS-9), APC-conjugated mAb to FoxP3 (clone 236A/E7), and Fixable Vibility Dye eFluor^®^ 506 were from eBioscience (Paris, France). PE-conjugated mAb to CD40 (clone MAB89) was from Beckman Coulter (Villepinte, France). Blocking mAb to human PD-L1 (clone MIH1) and isotype control mAb were from eBioscience. Alexa-488 conjugated mAb to IL-10 (clone JES59D7) and blocking mAb to PD-1 (clone EH12.2H7) were from Biolegend (London, UK).

### *M. tuberculosis* Antigens

γ-irradiated *M. tuberculosis* (strain H37Rv) and *M. tuberculosis* cell wall, cell membrane cytoplasmic fractions were obtained from BEI resources NIAID, NIH.

### Purification of Immune Cells

Peripheral blood mononuclear cells (PBMCs) were obtained from buffy bags of healthy donors by Ficoll density gradient centrifugation. Buffy bags of the healthy blood donors were purchased from Centre Necker-Cabanel, Etablissement Français du Sang, Paris, France. Ethical committee permission was obtained for the use of buffy bags of healthy donors (Institut National de la Santé et de la Recherche-EFS ethical committee convention 15/EFS/012). Monocytes and autologous CD4^+^ T cells were isolated from PBMCs by positive selection using the human CD14 and the CD4 MicroBeads (Miltenyi Biotec, Paris, France), respectively. The cell purity was more than 97%.

### Generation of DCs

Monocytes (0.5 × 10^6^ cells/ml) were cultured in the presence of granulocyte-macrophage colony-stimulating factor (GM-CSF; 1,000 IU/10^6^ cells) and IL-4 (500 IU/10^6^ cells) (both cytokines from Miltenyi Biotec) for 5 days to obtain immature monocyte-derived DCs (38). The differentiation of DCs was confirmed by flow cytometry.

### Stimulation of Monocytes and DCs with *M. tuberculosis* and Their Fractions

Monocytes or DCs (0.5 × 10^6^/ml) were cultured with (20 μg/ml) γ-irradiated *M. tuberculosis* or *M. tuberculosis*-derived cell wall, cell membrane, or cytoplasmic fractions (10 μg/ml) for 24 h. Activation of DCs and monocytes was assessed based on the expression of HLA-DR, CD40, CD80, and CD86 by using fluorescence-conjugated mAbs. In addition, induction of PD-L1 and PD-L2 on these cells was also analyzed.

### Monocyte–CD4^+^ T Cell and DC–CD4^+^ T Cell Cocultures

Monocytes or DCs (10,000 cells/200 μl/well) were cocultured with autologous CD4^+^ T cells at a ratio of 1:10 in U-bottom 96-well plates and stimulated with (20 μg/ml) γ-irradiated *M. tuberculosis* or *M. tuberculosis-*derived cell wall, cell membrane, or cytoplasmic fractions (10 μg/ml) for 5 days. After 5 days, cell-free supernatants were collected, and T cells were activated with phorbol myristate acetate (50 ng/ml) and ionomycin (500 ng/ml, Sigma-Aldrich, France), along with GolgiStop (BD Biosciences), for 4 h. For the analysis of IL-17^+^CD4^+^ T cells and IL-10^+^CD4^+^ T cells in the cocultures, surface staining was performed with fluorescence-conjugated mAbs to CD4. Then, cells were fixed, permeabilized using intracellular staining kit (eBioscience), and incubated at 4°C with fluorescence-conjugated mAbs to IL-17A, IL-10, pSTAT3, and RORC. Samples were acquired by using LSR II (BD Biosciences) flow cytometry, and data were analyzed by BD FACS DIVA software (BD Biosciences).

For the analysis of FoxP3^+^CD4^+^ T in the cocultures, surface staining was performed with fluorescence-conjugated mAb to CD4. Then, cells were fixed, permeabilized using intracellular staining kit (eBioscience), and incubated at 4°C with fluorescence-conjugated mAb to FoxP3.

For the analysis of PD-1 on CD4^+^ T cells, surface staining was performed with fluorescence-conjugated mAbs to CD4 and PD-1.

### PD-L1 and PD-1 Blocking Experiment

Autologous monocyte–CD4^+^ T cell and DC–CD4^+^ T cell cocultures were stimulated with *M. tuberculosis* for 18 h. Anti-PD-L1 (10 μg/ml), anti-PD-1 (10 μg/ml), or isotype control mAbs were then added to the coculture. After 5 days, frequency of IL-17A^+^CD4^+^ T cells and IL-17 secretion were analyzed.

### Validation of Role for Innate Cytokines in *M. tuberculosis*-Mediated Th17 Responses

Dendritic cells (10,000 cells/200 μl/well) were cocultured with autologous CD4^+^ T cells at a ratio of 1:10 in U-bottom 96-well plates and stimulated with (20 μg/ml) γ-irradiated *M. tuberculosis* or *M. tuberculosis*-derived cytoplasmic fractions (10 μg/ml) either alone or in the presence of (10 ng/ml) rhIL-1β (R&D systems, Lille, France) or rhIL-23 (PeproTech, Neuilly-Sur-Seine, France) for 5 days. After 5 days, cell-free supernatants were collected, and T cells were analyzed for Th17 responses by intracellular staining as described earlier.

### Quantification of Cytokines

IL-17A, IFN-γ, IL-6, IL-1β, and IL-23 in the cell-free supernatants of monocyte–CD4^+^ T cell and DC–CD4^+^ T cell cocultures were quantified by ELISA (Ready-SET-Go, eBioscience).

### Statistical Analysis

Statistical analyses were performed by two-way non-parametric Mann–Whitney test or one-way ANOVA (Kruskal–Wallis test or Holm–Sidak’s multiple comparisons test) as indicated using Prism 6 software. *P* < 0.05 was considered significant.

## Results

### Human Monocytes Promote Th17 Response to *M. tuberculosis*

**Figure 1:**
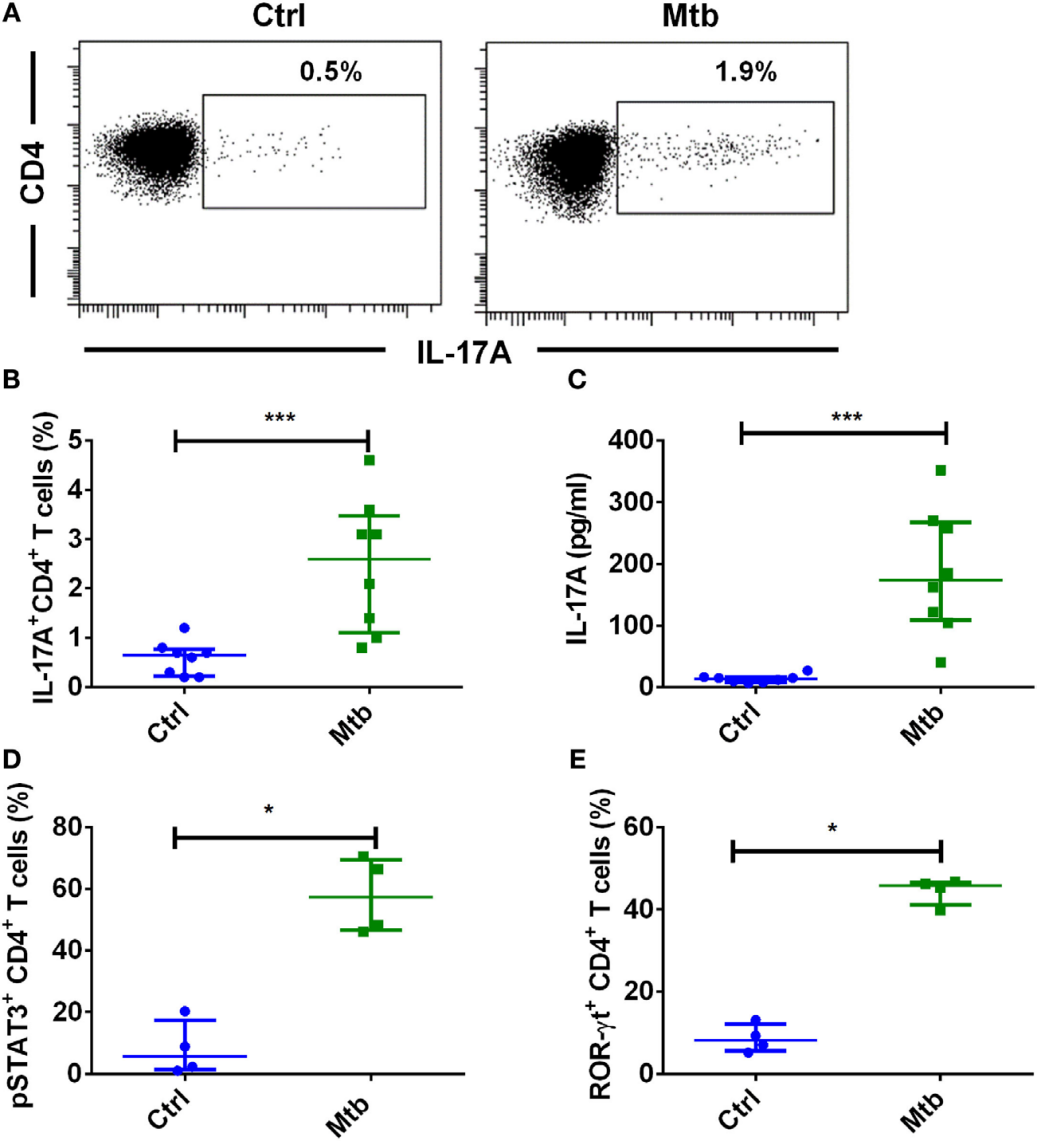
**Human monocytes stimulated with *M. tuberculosis* enhance Th17 response**. Human peripheral blood monocytes were cocultured with autologous CD4^+^ T cells at a ratio of 1:10 in X-vivo medium for 5 days with or without γ-irradiated *M. tuberculosis* (Mtb). Th17 cells were analyzed by flow cytometry by combination of surface staining for CD4 and intracellular staining for IL-17A, pSTAT3, and ROR-γt. IL-17A in the cell-free supernatants was quantified by ELISA. **(A,B)** Representative dot plots showing the frequencies of CD4^+^IL-17A^+^ T cells and **(B)** median ± SEM data from eight donors. **(C)** The amount of secretion of IL-17A (median ± SEM, *n* = 8). **(D)** Percentage of CD4^+^ T cells positive for pSTAT3 (median ± SEM, *n* = 4). **(E)** Percentage of CD4^+^ T cells positive for ROR-γt (median ± SEM, *n* = 4). *P < 0.05; ***P < 0.001; as determined by Mann–Whitney test.

Tuberculosis is associated with an expansion of immunomodulatory CD16^+^ monocyte population (39). Monocytes have been implicated in the establishment of Th17 response in autoimmune diseases (40, 41) and infection (42). We first investigated the ability of human monocytes to promote Th17 response to *M. tuberculosis*. We found that *M. tuberculosis*-stimulated monocytes significantly enhanced both frequency of IL-17A^+^CD4^+^ T cells (Figures 1A,B) and the amount of secretion of IL-17A (Figure 1C). On further examination of downstream Th17 signaling events in CD4^+^ T cells, we found that *M. tuberculosis*-stimulated monocytes significantly enhanced the frequency of pSTAT3 (Figure 1D) and RORC (Figure 1E). These results thus suggest that human monocytes have the ability to promote Th17 response to *M. tuberculosis*.

### Different Antigen Fractions of *M. tuberculosis* Have Similar Capacity to Induce Monocyte-Mediated Th17 Response

**Figure 2:**
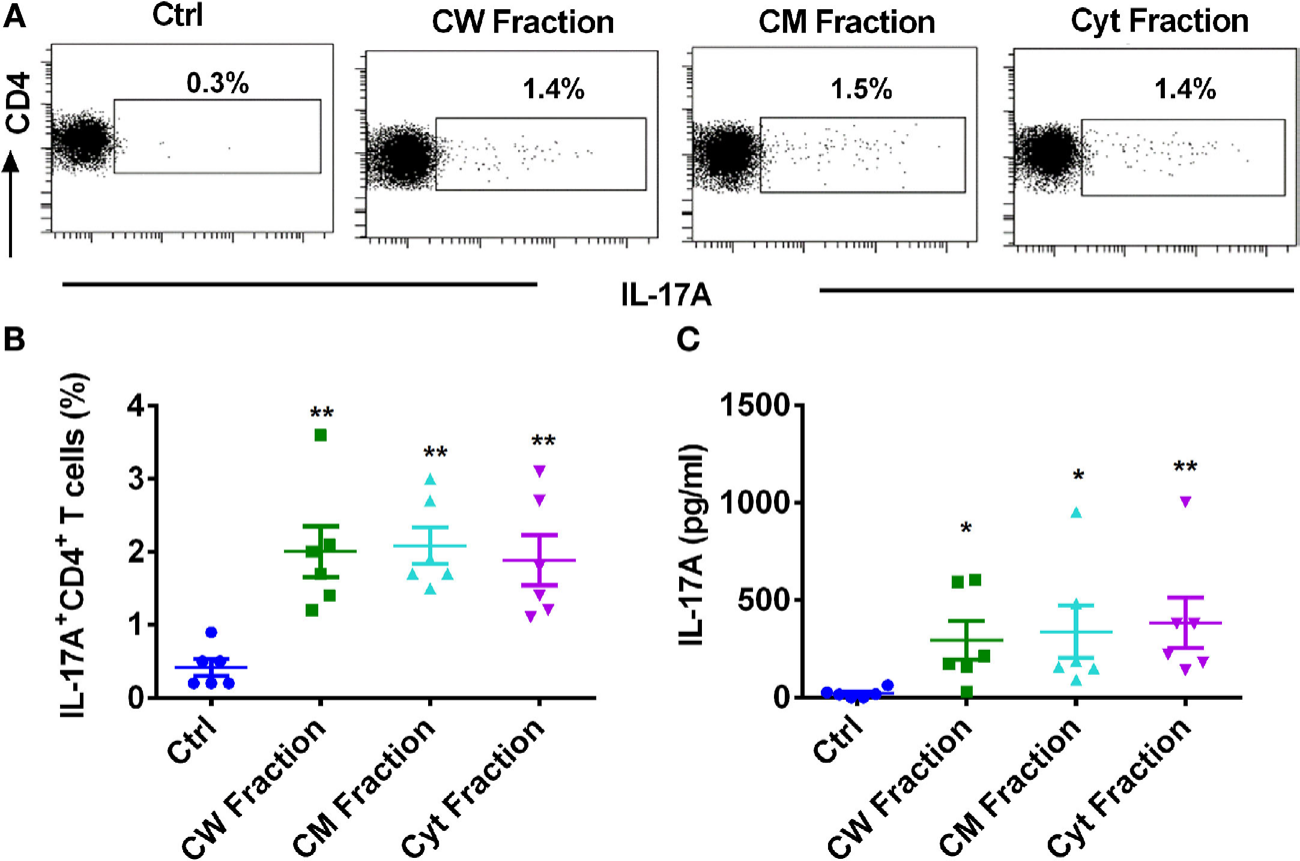
**Different antigen fractions of *M. tuberculosis* have similar capacity to amplify human monocyte-mediated Th17 response**. Monocytes were cocultured with autologous CD4^+^ T cells either alone or were stimulated with *M. tuberculosis*-derived cell wall (CW), cell membrane (CM), or cytoplasmic (Cyt) fractions for 5 days. Th17 cells were analyzed by flow cytometry by combination of surface staining for CD4 and intracellular staining for IL-17A. IL-17A in the cell-free supernatants was quantified by ELISA. **(A,B)** Representative dot plots showing the frequencies of CD4^+^IL-17A^+^ T cells and **(B)** mean ± SEM data from six independent donors. **(C)** The amount of secretion of IL-17A (mean ± SEM, *n* = 6). *P < 0.05; **P < 0.01; as determined by one-way ANOVA.

*Mycobacterium tuberculosis* possesses a plethora of antigens to modulate immune response. Most of these antigens are either located in the cell wall, cell membrane, or cytosol. Hence, we investigated whether these different antigens of *M. tuberculosis* have similar or distinct ability to mount monocyte-mediated Th17 response. All these antigenic fractions induced similar level of activation of monocytes as shown by the significantly augmented expressions of CD80, CD86, and CD40 (Figure S1 in Supplementary Material). Consistent with the activation status of monocytes, all the antigen fractions, i.e., cell wall, cell membrane, and cytoplasmic fractions significantly enhanced the frequency of IL-17A^+^CD4^+^ T cells (Figures 2A,B) and the secretion of IL-17A (Figure 2C). However, we observed no significant differences in the extent of Th17 response mediated by different fractions of *M. tuberculosis*. Our data thus indicate that all the antigen fractions of *M. tuberculosis* have similar ability to promote monocyte-mediated Th17 responses.

### Dendritic Cells Differentially Promote Th17 Response to *M. tuberculosis* and Its Antigen Fractions

**Figure 3:**
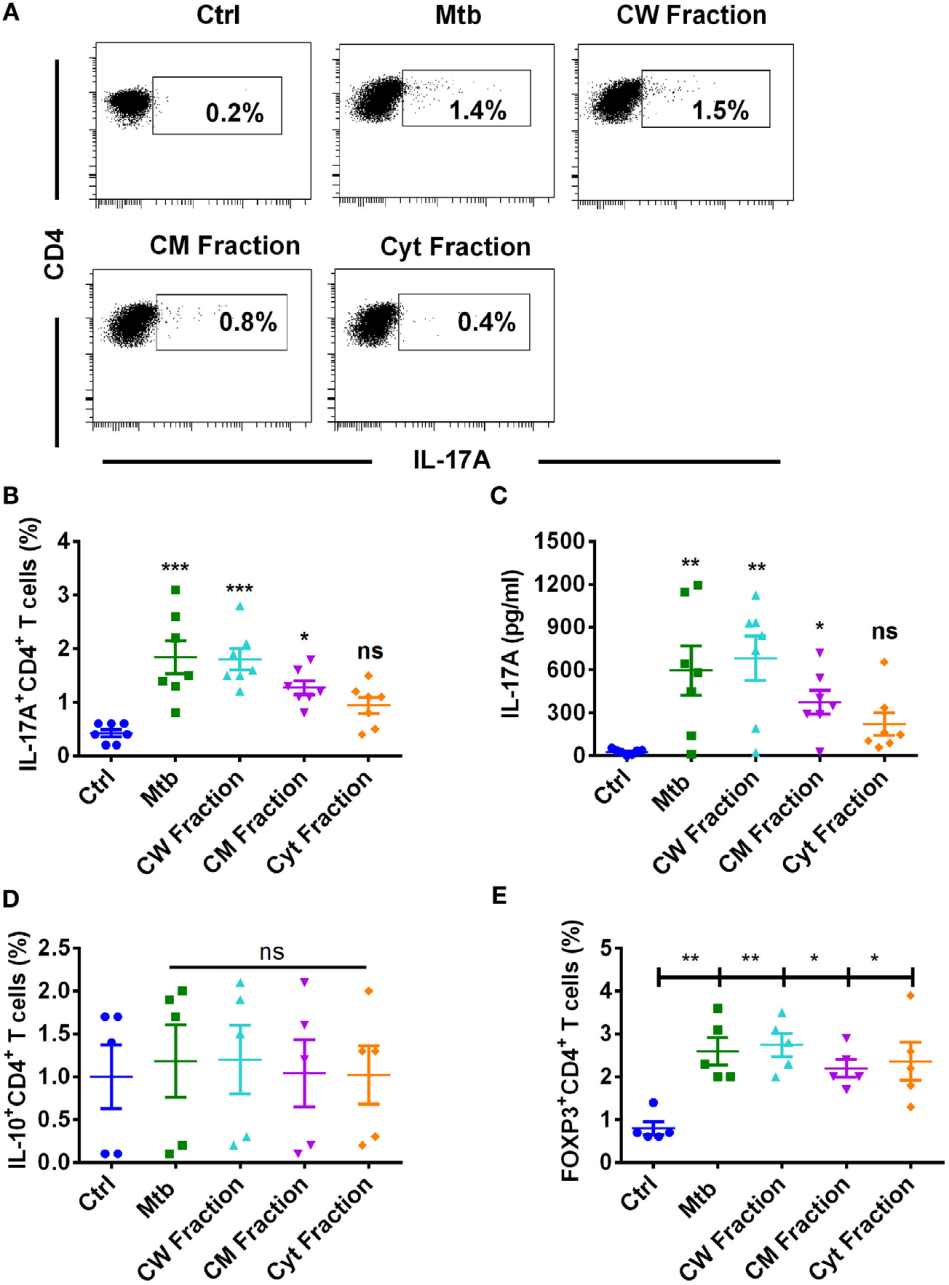
**Human dendritic cells differentially promote Th17 response to *M. tuberculosis* and its antigen fractions**. Human monocyte-derived DCs were cocultured with autologous CD4^+^ T cells at a ratio of 1:10 in X-vivo medium alone or with γ-irradiated *M. tuberculosis* (Mtb) or *M. tuberculosis*-derived cell wall (CW), cell membrane (CM), or cytoplasmic (Cyt) fractions for 5 days. Th17 cells were analyzed by flow cytometry by combination of surface staining for CD4 and intracellular staining for IL-17A. IL-17A in the cell-free supernatants was quantified by ELISA. **(A,B)** Representative dot plots showing the frequencies of CD4^+^IL-17A^+^ T cells and **(B)** mean ± SEM data from seven independent donors. **(C)** The amount of secretion of IL-17A (mean ± SEM, *n* = 7). **(D,E)** Frequencies of IL-10^+^CD4^+^ T cells or FoxP3^+^CD4^+^ Treg cells in DC–T cell cocultures stimulated with various antigens of *M. tuberculosis* (mean ± SEM, *n* = 5). *P < 0.05; **P < 0.01; ***P < 0.001; ns, not significant; as determined by one-way ANOVA.

Dendritic cells play a critical role in mediating protection to *M. tuberculosis* by priming T cell response. Hence, we investigated the capacity of DCs to promote Th17 response to *M. tuberculosis* and its antigen fractions. Our results indicate that DCs have the capacity to enhance Th17 response to *M. tuberculosis* (Figures 3A–C). However, in contrast to monocytes, human DCs displayed differential ability in stimulating Th17 response to different antigen fractions of *M. tuberculosis*. Thus, cell wall antigen fraction substantially enhanced Th17 response and was comparable to that induced by *M. tuberculosis* bacteria (Figures 3A–C). Although cell membrane fraction also enhanced Th17 response, it was lower than that observed with *M. tuberculosis* bacteria and cell wall fraction. Surprisingly, cytoplasmic fraction did not significantly enhance either frequencies of IL-17A^+^CD4^+^ T cells or the production of IL-17A (Figures 3A–C).

We confirm that low-level induction of Th17 response by cytoplasmic fraction was not because of lack of induction of DC maturation. In fact, DCs stimulated with cytoplasmic fraction significantly enhanced DC maturation markers HLA-DR, CD80, CD86, and CD40 and was similar to those observed with *M. tuberculosis* bacteria, cell wall, and cell membrane fractions (Figure S2 in Supplementary Material).

Further, the inability of cytoplasmic fraction to promote DC-mediated Th17 response was not due to increased frequency of suppressor T cells, such as IL-10^+^CD4^+^ T cells or FoxP3^+^CD4^+^ Treg cells. In fact, the frequency of IL-10^+^CD4^+^ T cells remain unaltered upon stimulation with *M. tuberculosis* or its antigen fractions (Figure 3D). Consistent with the previous findings, *M. tuberculosis* and its antigen fractions including cytoplasmic fraction significantly enhanced the frequency of FoxP3^+^CD4^+^ Tregs (Figure 3E). However, the extent of stimulation of Treg response was similar among all three antigen fractions of *M. tuberculosis*.

### Differential Expression of PD-L1 on Monocytes and DCs upon Stimulation with *M. tuberculosis* and Its Antigen Fractions

**Figure 4:**
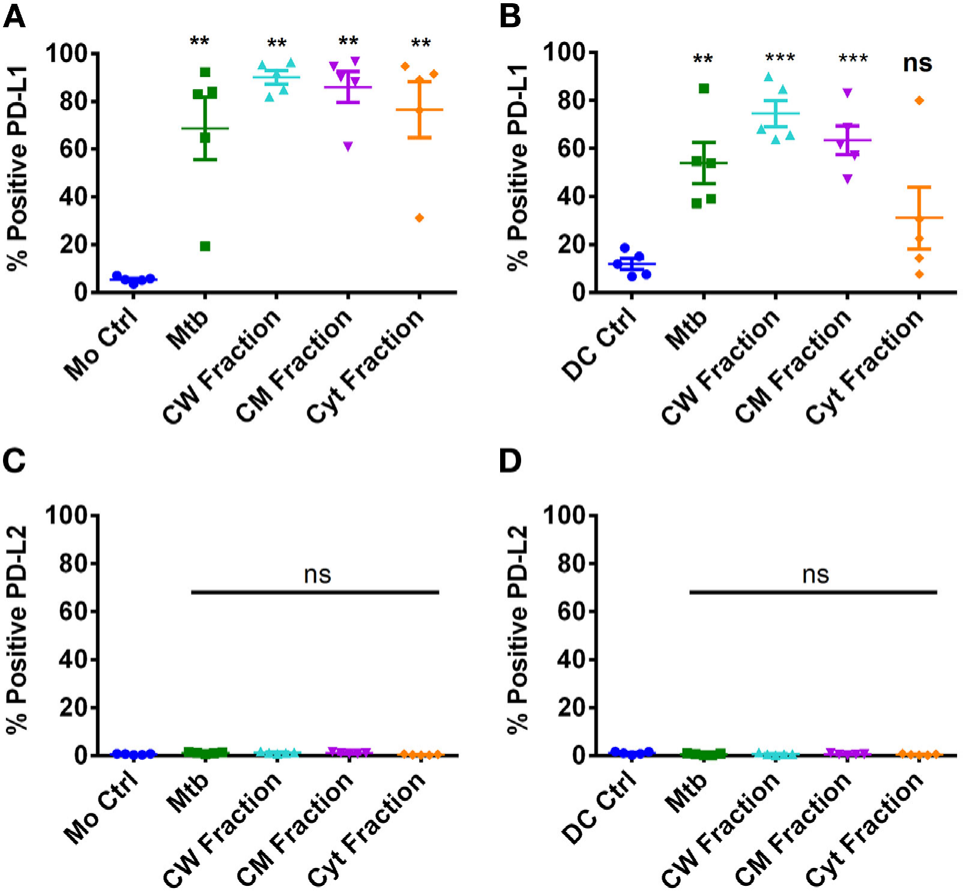
**Differential expression of PD-L1 and PD-L2 on monocytes and dendritic cells upon stimulation with *M. tuberculosis* and its antigen fractions**. Monocytes or monocyte-derived DCs were either cultured alone or stimulated with γ-irradiated *M. tuberculosis* or *M. tuberculosis*-derived cell wall (CW), cell membrane (CM), or cytoplasmic (Cyt) fractions for 24 h. Surface expressions of PD-L1 **(A,B)** and PD-L2 **(C,D)** were analyzed by flow cytometry. **(A,C)** Percentage of monocytes expressing PD-L1 and PD-L2 (mean ± SEM, *n* = 5). **(B,D)** Percentage of DCs expressing PD-L1 and PD-L2 (mean ± SEM, *n* = 5). **P < 0.01; ***P < 0.001; ns, not significant; as determined by one-way ANOVA.

*M. tuberculosis* employs several strategies to modulate effector CD4 T cell response (43). Several recent reports have indicated that PD-1–PD-L1/PD-L2 axis has a pivotal role in the regulation of T cell response to *M. tuberculosis* (29, 31, 33). Therefore, to decipher the role of PD-L1/PD-L2 in regulating Th17 response to *M. tuberculosis*, we first investigated the ability of *M. tuberculosis* and its different antigen fractions in modulating PD-L1 and PD-L2 expression on monocytes and DCs. Our results reveal that *M. tuberculosis* bacteria, cell wall fraction, and cell membrane fractions significantly induce PD-L1 expression on both monocytes and DCs (Figures 4A,B). However, significant induction of PD-L1 by cytoplasmic fraction was observed only in monocytes but not in DCs (Figures 4A,B). Unstimulated monocytes and DCs did not express PD-L2 and was not altered upon stimulation with either *M. tuberculosis* or its different antigen fractions (Figures 4C,D).

### PD-L1 on Human Innate Cells Regulates Th1, But Not Th17, Response to *M. tuberculosis*

**Figure 5:**
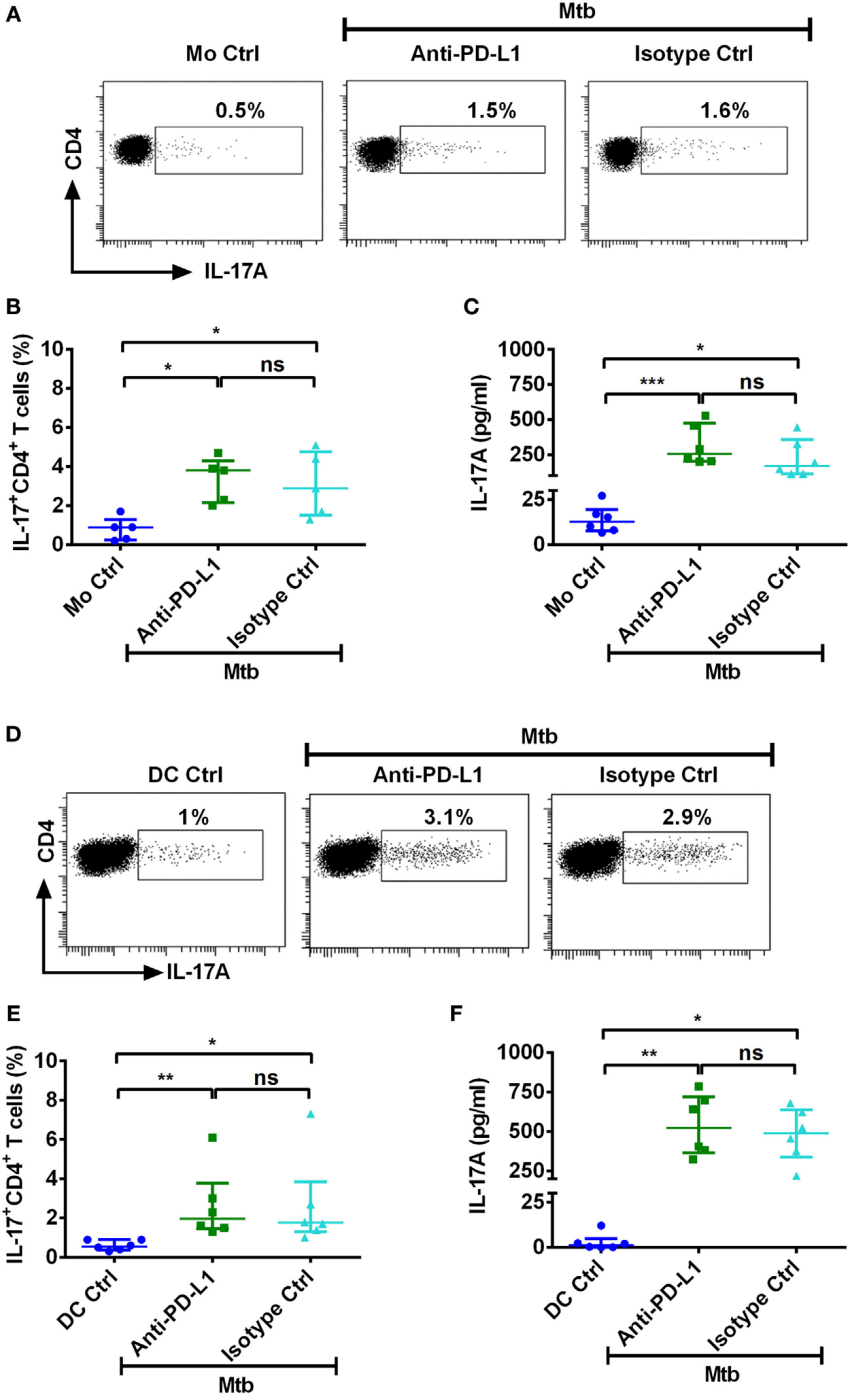
**PD-L1 on human innate cells is dispensable for regulating Th17 response to *M. tuberculosis***. Monocytes or DCs were cocultured with autologous CD4^+^ T cells either alone or with γ-irradiated *M. tuberculosis*. After 18 h, PD-L1 blocking mAb or isotype control mAb were added, and cultures were maintained for 5 days. Th17 cells were analyzed by flow cytometry by combination of surface staining for CD4 and intracellular staining for IL-17A. IL-17A in the cell-free supernatants was quantified by ELISA. **(A–C)** Representative dot plots showing the frequencies of CD4^+^IL-17A^+^ T cells **(A)**, frequencies of CD4^+^IL-17A^+^ T cells (mean ± SEM, *n* = 5) **(B)**, and amounts of IL-17A production (mean ± SEM, *n* = 6) **(C)** in monocyte–CD4^+^ T cell cocultures. **(D–F)** Representative dot plots showing the frequencies of CD4^+^IL-17A^+^ T cells **(D)**, frequencies of CD4^+^IL-17A^+^ T cells (mean ± SEM, *n* = 6) **(E)**, and amounts of IL-17A production (mean ± SEM, *n* = 6) **(F)** in DC–CD4^+^ T cell cocultures. *P < 0.05; **P < 0.01; ***P < 0.001; ns, not significant; as determined by one-way ANOVA.

PD-1–PD-L1 interaction has been reported to inhibit Th1 response to *M. tuberculosis*. To explore if PD-L1 also checks Th17 response to *M. tuberculosis*, we employed blocking antibodies to PD-L1. We found that blocking PD-L1 either in the monocyte–CD4^+^ T cell coculture or in the DC–CD4^+^ T cell coculture did not significantly modify either the frequencies of IL-17A^+^CD4^+^ T cells or the secretion of IL-17A (Figures 5A–F). These data are in line with the fact that lack of induction of PD-L1 on DCs by cytoplasmic fraction of *M. tuberculosis* was not associated with enhanced Th17 responses (Figures 3 and 4B). Whereas, same cytoplasmic fraction promoted Th17 response despite it induced significant expression of PD-L1 on monocytes (Figures 2 and 4A). Taken together, these results imply that PD-L1 does not function as negative regulator of Th17 response to *M. tuberculosis*.

**Figure 6:**
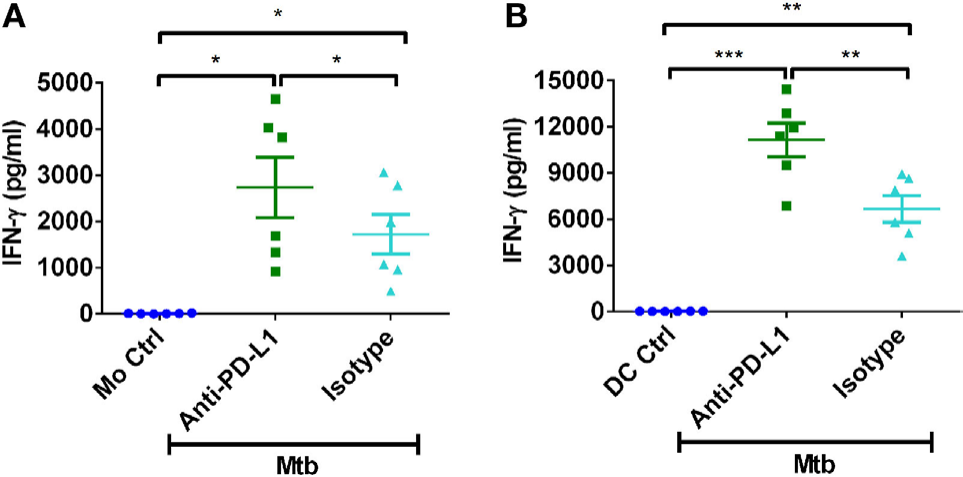
**PD-L1 on human innate cells regulates Th1 response to *M. tuberculosis***. Monocytes or DCs were cocultured with autologous CD4^+^ T cells either alone or with γ-irradiated *M. tuberculosis*. After 18 h, PD-L1 blocking mAb or isotype control mAb were added, and cultures were maintained for 5 days. Amounts of secretion of IFN-γ in monocyte–CD4^+^ T cell cocultures **(A)** and in DC–CD4^+^ T cell cocultures (mean ± SEM, *n* = 6) **(B)**. *P < 0.05; **P < 0.01; ***P < 0.001; ns, not significant; as determined by one-way ANOVA.

It was important to demonstrate that observed phenomenon is not due to inefficient PD-L1 blocking. Previous results have demonstrated that PD-L1 negatively regulates IFN-γ responses. Therefore, to demonstrate the efficiency of PD-L1 blocking, we have assessed the secretion of IFN-γ in the monocyte–CD4^+^ T cell and DC–CD4^+^ T cell cocultures. Consistent with the previous data, blockade of PD-L1 significantly augmented the production of IFN-γ from CD4^+^ T cells (Figures 6A,B).

### PD-1 Is Dispensable for the Regulation of Th17 Response to *M. tuberculosis*

**Figure 7:**
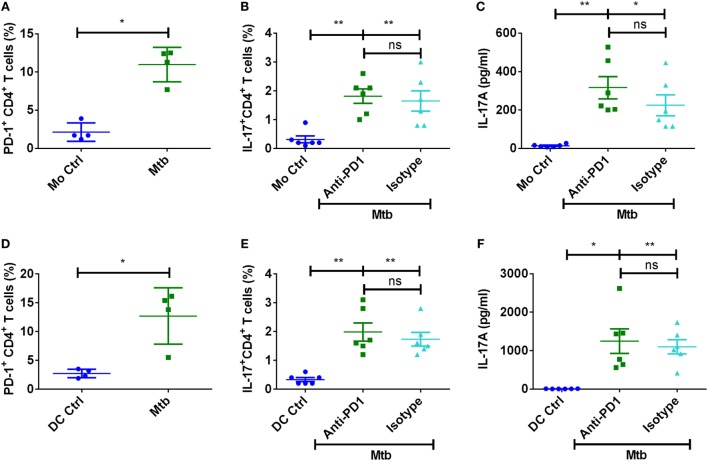
**PD-1 is dispensable for the regulation of Th17 response to *M. tuberculosis***. Monocytes or DCs were cocultured with autologous CD4^+^ T cells either alone or with γ-irradiated *M. tuberculosis*. After 18 h, PD-1 blocking mAb or isotype control mAb were added, and cultures were maintained for 5 days. **(A–C)** Frequency of PD-1^+^CD4^+^ T cells (mean ± SEM, *n* = 4) **(A)**, frequency of CD4^+^IL-17A^+^ T cells (mean ± SEM, *n* = 6) **(B)**, and amounts of IL-17A production (mean ± SEM, *n* = 6) **(C)** in the monocyte–CD4^+^ T cell cocultures. **(D–F)** Frequency of PD-1^+^CD4^+^ T cells (mean ± SEM, *n* = 4) **(D)**, frequency of CD4^+^IL-17A^+^ T cells (mean ± SEM, *n* = 6) **(E)**, and amounts of IL-17A production (mean ± SEM, *n* = 6) **(F)** in the DC–CD4^+^ T cell cocultures. *P < 0.05; **P < 0.01; ns, not significant; as determined by one-way ANOVA.

Programed death ligand-1 signals *via* PD-1 on CD4^+^ T cells, and recently it was reported that *Mycobacterium*-induced PD-1 coordinates suppression of Th17 response in tuberculosis patients (28). We confirm that *M. tuberculosis* significantly enhances the frequency of PD-1^+^ T cells in the DC/monocyte–CD4^+^ T cell cocultures (Figures 7A,D). However, blockade of PD-1 did not significantly alter either the frequencies of IL-17A^+^CD4^+^ T cells or the secretion of IL-17A (Figures 7B,C,E,F). Similar to PD-L1 experiments, blockade of PD-1 also lead to increased IFN-γ responses. Together, these data indicate that human Th17 response to *M. tuberculosis* is not under the control of PD pathway.

### IL-1β Is Critical for Mediating Th17 Response to *M. tuberculosis*

**Figure 8:**
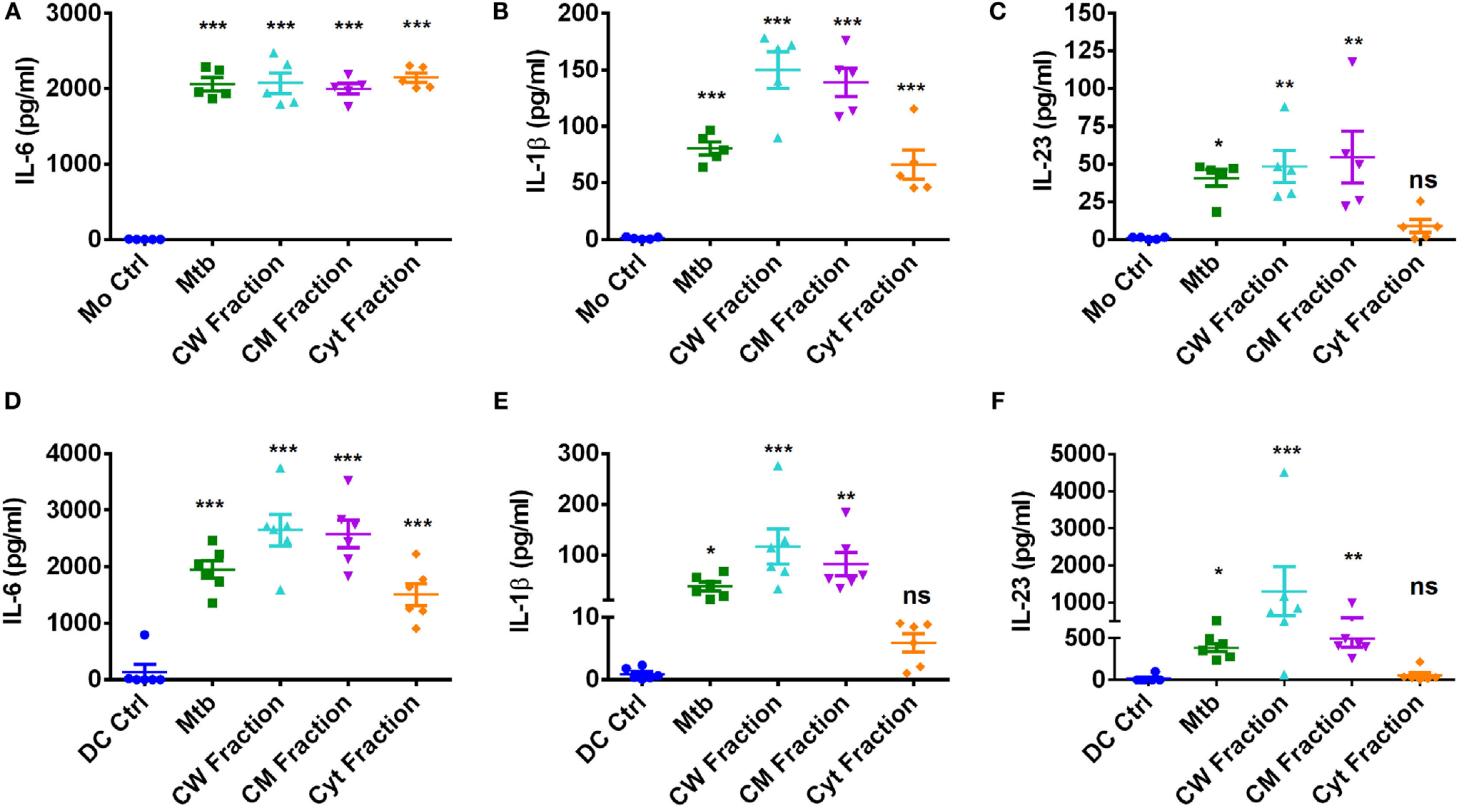
**Production of Th17 priming cytokines by human innate cells upon stimulation with *M. tuberculosis* or its antigen fractions**. Monocytes or DCs were cocultured with autologous CD4^+^ T cells either alone or stimulated with γ-irradiated *M. tuberculosis* or *M. tuberculosis*-derived cell wall (CW), cell membrane (CM), or cytoplasmic (Cyt) fractions for 5 days. Cell-free supernatants were analyzed for the secretion of innate cytokines by ELISA. **(A–C)** IL-6, IL-1β, and IL-23 production by monocytes (mean ± SEM, *n* = 5) and **(D–F)** IL-6, IL-1β, and IL-23 production by DCs (mean ± SEM, *n* = 6). *P < 0.05; **P < 0.01; ***P < 0.001; ns, not significant; as determined by one-way ANOVA.

Upon encountering with pathogens, APCs secrete various cytokines that dictate the outcome of T cell response. Particularly, IL-6, IL-1β, and IL-23 derived from innate immune cells play a crucial role in establishing and sustaining Th17 response. In addition, IL-21 and TGF-β also have important role in Th17 priming (8, 14). Thus, to decipher the mechanism of differential Th17 response to *M. tuberculosis* and its fractions by monocytes and DCs, we analyzed the Th17-polarizing cytokines secreted by these innate cells. IL-21 was not produced by monocytes and DCs upon stimulation with *M. tuberculosis* and its antigen fractions. Similarly, basal level of TGF-β was not significantly altered by *M. tuberculosis*. However, upon stimulation with *M. tuberculosis* and its antigen fractions, both innate cells secreted large quantities of IL-6 (Figures 8A,D) and moderate amounts of IL-1β (Figures 8B,E). Of note, monocytes produced significantly lower amounts (~10-fold) of IL-23 as compared to DCs (Figures 8C,F). Interestingly, consistent with the lack of role in the modulation of Th17 response, none of these innate cytokines were significantly altered upon blockade of PD-L1 (Figure S3 in Supplementary Material).

**Figure 9:**
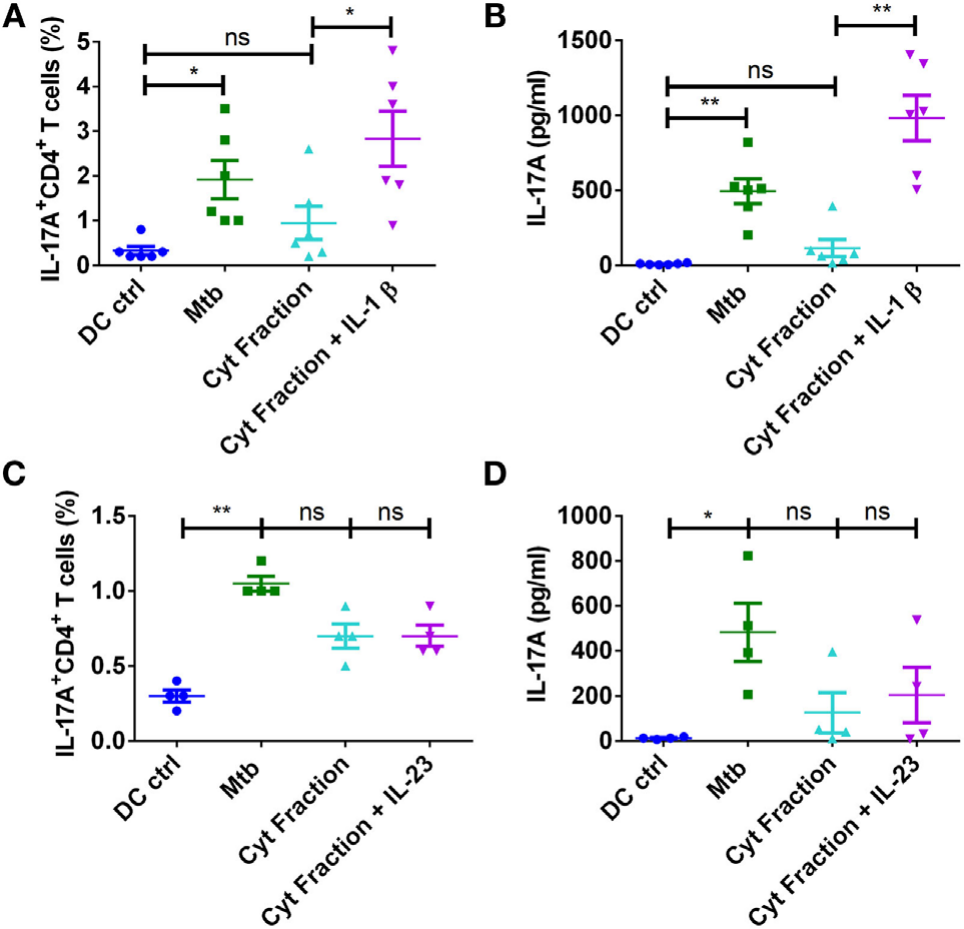
**IL-1β is critical for mediating Th17 response to *M. tuberculosis***. DCs were cocultured with autologous CD4^+^ T cells either alone or stimulated with γ-irradiated *M. tuberculosis, M. tuberculosis*-derived cytoplasmic (Cyt) fraction, or Cyt fraction in combination with exogenous IL-1β **(A,B)** or IL-23 **(C,D)** for 5 days. Frequencies of CD4^+^IL-17A^+^ T cells (mean ± SEM, *n* = 4–6) **(A,C)** and amounts of IL-17A production (mean ± SEM, *n* = 4–6) **(B,D)** in DC–CD4^+^ T cell cocultures. *P < 0.05; **P < 0.01; ns, not significant; as determined by one-way ANOVA.

Despite induction of large quantities of IL-6 in DCs similar to those induced by *M. tuberculosis* or its other antigen fractions, lack of Th17 response by cytoplasmic fraction provide a pointer toward dispensability of this cytokine in *M. tuberculosis*-mediated Th17 response. The similar amount of IL-1β production by stimulated monocytes explains elicitation of Th17 response by all the antigen fractions of *M. tuberculosis*. On the contrary, cytoplasmic fraction was inferior to cell wall and cell membrane antigen fractions in promoting IL-1β and IL-23 by DCs, and this could explain the lack of Th17 responses observed in DCs upon stimulation with cytoplasmic fraction. Therefore, to establish a role for IL-1β and IL-23 in *M. tuberculosis*-mediated Th17 responses, we supplemented IL-1β or IL-23 to the DC–CD4^+^ T cell cocultures that are stimulated with cytoplasmic fraction of *M. tuberculosis*. We found that supplementation of IL-1β enabled cytoplasmic fraction-stimulated DCs to mount strong Th17 response (Figures 9A,B), while IL-23 had no effect (Figures 9C,D). Our results thus reveal that innate Th17-polarizing cytokine IL-1β but not members of the PD pathway. dictates the outcome of human Th17 response to *M. tuberculosis* and its antigen fractions.

## Discussion

*M. tuberculosis*-loaded aerosols that enter the lungs interact with resident phagocytes, which include alveolar macrophages and DCs. Different subpopulations of DCs are known to coexist in the human lungs (44). Circulating monocytes form a major reservoir for tissue macrophages and different subsets of DCs (45–47). Moreover, monocytes are known to give rise to DCs *in vivo* at mucosal surfaces, such as skin (48) and lungs (49, 50), thus suggesting an *in vivo* relevance of monocyte-derived DCs in mediating immune response to mucosal pathogens, such as *M. tuberculosis*. In comparison to other infections, there is a delay in the onset of adaptive immune response upon *M. tuberculosis* infection allowing the bacteria to form a niche in the lungs (51). While it is clear that Th1/IFN-γ response is indispensable for the protection against tuberculosis, the role of Th17/IL-17 in mediating protection is unclear.

Initial studies in mice suggested that the IL-23/Th17 axis is dispensable for the overall protection against *M. tuberculosis* challenge but is required for enhancing the formation of granuloma in the absence of an active IL-12/Th1 axis (52). Surprisingly, Th17 response mediated protection to a highly virulent *M. tuberculosis* strain HN878 by activating macrophages and curtailing bacterial burden in the lungs. However, it was dispensable to the less virulent strains of *M. tuberculosis* thus ascribing a protective role for Th17 to emerging virulent strains (53). Earlier reports in tuberculosis patients indicated that virulent strains, such as multi-drug resistant *M. tuberculosis*, strongly induce Th17 response. However, enhanced IL-17 was correlated to the severity of the disease and high bacterial burden in the lungs, suggesting a detrimental role for Th17 response in humans (54). More recent reports have demonstrated that tuberculosis patients display lower antigen-specific Th17 response. Incidentally, anti-tuberculosis therapy not only enhanced Th1 response but also augmented Th17 response (28). Furthermore, individuals with biallelic RORC mutations with a defective Th17 response are associated with a compromised Th1 response and are susceptible to fungal and mycobacterial infections (55). Thus, it appears that the protective roles of Th17 response may vary depending on the stage of infection. Th17 response contributes to vaccine-mediated protection and during the earlier stages of infection by recruiting lymphocytes and promoting Th1 response. However, chronic exposure or during the later stages of infection it may be detrimental due to neutrophil recruitment that can mediate tissue damage leading to immunopathology.

Previous studies have shown that DCs can mediate Th17 response to *M. tuberculosis* by signaling through TLR-2, dectin-1, DC-SIGN, and mannose receptors (36, 53, 56). In the present study, we show that monocytes and DCs have differential capacity to modulate Th17 responses to *M. tuberculosis* and *M. tuberculosis*-derived antigen fractions. Notably, DCs evoked a much stronger IL-17A production from CD4^+^ T cells than monocytes. This might be due to the capacity of DCs to secrete large quantities of IL-23 than monocytes. IL-23 plays an important role in stabilizing and sustaining the ensuing Th17 response. Interestingly, unlike monocytes that promoted Th17 response to cell wall, cell membrane, and cytoplasmic fractions of *M. tuberculosis*, DCs displayed differential response to these antigen fractions. Thus, cell wall fraction triggered strong DC-mediated IL-17A production from CD4^+^ T cells; cell membrane fraction promoted intermediate IL-17A response, and the cytoplasmic fraction did not significantly modulate Th17 response. As immuno suppressor T cells have the ability to inhibit effector T cell responses, we surmised whether increased frequency of IL-10^+^CD4^+^ T cells or FoxP3^+^CD4^+^ Treg cells was responsible for low-level induction of DC-mediated Th17 responses by cytoplasmic fraction of *M. tuberculosis* (57–60). However, IL-10^+^CD4^+^ T cells were not induced under any stimulatory conditions. Although cytoplasmic fraction significantly enhanced the frequency of FoxP3^+^CD4^+^ Treg cells, this increase was similar to that observed with *M. tuberculosis* and other antigen fractions.

Suppression of cellular immunity reckons a major evasion strategy employed by *M. tuberculosis*. Of the numerous evasion strategies employed by *M. tuberculosis*, the exploitation of PD-L1/PD-L2–PD-1 axis occupies a central role due to its implication in the expansion of Treg response and suppression of effector Th1 response (61, 62). Considering that induced Tregs and Th17 responses are reciprocally regulated, the possible role of PD-L1/PD-L2 in regulating Th17 response has not been questioned. It is well established that *M. tuberculosis* infection induces PD-L1 on APCs and PD-1 on T cells. Interaction of PD-L1 with PD-1 promotes Treg differentiation and expansion by activating SHP1/2. Induced SHP1/2 suppresses STAT1 functions, abrogates IFN-γ production, and abolishes its inhibitory effect on FOXP3 leading to Treg expansion (63). Since PD-1 axis plays a critical role in mediating immune tolerance and loss of which can predispose to inflammatory conditions and autoimmune diseases (64, 65), it is deleterious to completely abrogate PD-L1–PD-1 signaling during *M. tuberculosis* infection. This is evidenced by the fact that PD-L1-deficient and PD-1-deficient mice are susceptible to *M. tuberculosis* infection and display exacerbated inflammation (66, 67).

In the present study, we found that monocytes stimulated with *M. tuberculosis* and its antigen fractions significantly induce PD-L1 expression on human monocytes. On the contrary, only cell wall and cell membrane fractions induced PD-L1 expression on DCs, whereas the cytoplasmic fraction failed to enhance PD-L1 on DCs. PD-L2 was not expressed on both monocytes and DCs under any stimulatory conditions. The inability of cytoplasmic fraction to induce PD-L1 on DCs was not due to its lack of stimulatory capacity as cytoplasmic fraction inducted DC maturation similar to that of cell wall and cell membrane fractions.

To decipher the role PD-L1 in regulating Th17 response to *M. tuberculosis*, we employed blocking antibodies to PD-L1. Our results demonstrate that PD-L1 blockade did not significantly alter the frequencies of Th17 cells. On the other hand, IFN-γ production was significantly enhanced when PD-L1 was blocked, thus confirming the previous data on role of PD axis in regulating Th1 responses (31, 32). However, we did observe a marginal (~50 pg/ml) but insignificant increase in IL-17 production upon PD-L1 blockade. As Th17 cells and Tregs are reciprocally regulated and moreover Tregs are known to suppress Th17 response (66, 68, 69), it is likely that blockade of PD-L1 can indirectly favor Th17 response to a certain extent. This could explain minimal augmentation of IL-17A production that we observed upon PD-L1 blockade.

In addition to PD-L1, *M. tuberculosis* significantly enhanced PD-1 on CD4^+^ T cells. However, PD-1 blockade had no repercussion on either monocyte- or DC-mediated Th17 response. Our data contradict a recent report that indicated that *Mycobacterium*-induced PD-1 orchestrates suppression of Th17 response in tuberculosis patients (28). This discrepancy might be due to the fact that previous report focused on active disease where prolonged infection can enhance additional co-stimulatory molecules that might not be present during the earlier phases of stimulation. For example, APCs from active disease express both the co-stimulatory molecules PD-L1 and PD-L2 (31), whereas our current data and previous reports show that DCs from healthy individuals stimulated with *M. tuberculosis* both at the transcript level as well as the protein level induce only PD-L1 and not PD-L2 (30, 33). It is essential to note that we have used γ-irradiated *M. tuberculosis* or *M. tuberculosis*-derived cell wall, cell membrane, or cytoplasmic fractions in our experiments, and these conditions may not completely mimic *in vivo* situation of infection with live bacteria. Therefore, further work is necessary to decipher the role of PD-1–PD-L1/PD-L2 pathway in tuberculosis. Nevertheless, our report indicates that PD pathway does not contribute to negative regulation of human Th17 response to *M. tuberculosis*. Also, cytoplasmic fraction of *M. tuberculosis* failed to induce Th17 response despite lack of induction of PD-L1 on DCs.

To decipher the possible mechanism that explains inability of cytoplasmic fraction to promote DC-mediated Th17 response, we analyzed key Th17-polarizing cytokines secreted by monocytes and DCs. Our results indicate that cytoplasmic fraction failed to induce IL-1β, the key cytokine that promotes Th17 response. The amounts of secretion of IL-1β and Th17 responses were significantly correlated. Importantly, exogenous supplementation of IL-1β was sufficient to significantly augment Th17 response by the cytoplasmic fraction. These results indicate that inability of cytoplasmic fraction to induce the production of IL-1β from DCs resulted in its failure to prime a robust Th17 response. Further work on mechanisms underlying the differential production of IL-1β from monocytes and DCs upon stimulation with cytoplasmic fraction is warranted (70). Distinct expression of pattern-recognition receptors that sense cytoplasmic fraction on monocytes and DCs might have resulted in the differential IL-1β secretion upon stimulation with the cytoplasmic fraction. Therefore, antigens that come in direct contact with innate immune cells, such as the cell wall antigens, are attractive candidates for future tuberculosis vaccines. It is important to note that IL-1β is a critical mediator of immunity to *M. tuberculosis* (71, 72). Our results suggest that this function of IL-1β is in part *via* enhancement of Th17 responses. As tuberculosis patients were reported to exhibit lower antigen-specific Th17 response (28), supplementing IL-1β might enhance Th17 response.

Taken together, our report demonstrates that DCs have differential capacity to mediate Th17 response to various antigen fractions of *M. tuberculosis*. Therefore, cell wall antigens of *M. tuberculosis* constitute potential subunit vaccine candidates. Importantly, we demonstrate that PD pathway is critical for regulating Th1, but not Th17, response to *M. tuberculosis*. IL-1β however is necessary for promoting a prominent Th17 response to *M. tuberculosis*.

## Author Contributions

ES-V and JB designed the research. ES-V, VS, MD, AK, CS, ML, and CG performed the research. ES-V, VS, MD, AK, CS, ML, CG, SK, and JB contributed to data analyses and data interpretation. ES-V and JB wrote the manuscript. ES-V, VS, MD, AK, CS, ML, CG, SK, and JB revised the manuscript critically for important intellectual content and approved the final version.

## Conflict of Interest Statement

The authors declare that the research was conducted in the absence of any commercial or financial relationships that could be construed as a potential conflict of interest.

## Supplementary Material

Figure S1 ***M. tuberculosis* and its antigen fractions enhance costimulatory molecules on monocytes**. Monocytes (0.5 × 10^6^/ml) were either cultured alone or stimulated with γ-irradiated *M. tuberculosis* (Mtb) or *M. tuberculosis*-derived cell wall (CW), cell membrane (CM), or cytoplasmic (Cyt) fractions for 24 h. **(A–D)** Surface expression of CD80 [mean fluorescence intensity (MFI), CD86 (% positive cells), CD40 (MFI), and HLA-DR (MFI)] (mean ± SEM, *n* = 5) was analyzed by flow cytometry. ***P* < 0.01; ns, not significant; as determined by one-way ANOVA.

Figure S2 ***M. tuberculosis*-derived cytoplasmic fraction induce maturation of DCs**. DCs (0.5 × 10^6^/ml) were either cultured alone or stimulated with γ-irradiated *M. tuberculosis* (Mtb) or *M. tuberculosis*-derived cell wall (CW), cell membrane (CM), or cytoplasmic (Cyt) fractions for 24 h. **(A–D)** Surface expression of CD80 [mean fluorescence intensity (MFI), CD86 (% positive cells), CD40 (MFI), and HLA-DR (MFI)] (mean ± SEM, *n* = 4) was analyzed by flow cytometry. **P* < 0.05; ***P* < 0.01; ****P* < 0.001; as determined by one-way ANOVA.

Figure S3 **Production of Th17-polarizing cytokines by human innate cells upon PD-L1 blockade**. Monocytes or DCs were cocultured with autologous CD4^+^ T cells either alone or stimulated with γ-irradiated *M. tuberculosis* (Mtb). After 18 h, PD-L1 blocking mAb or isotype control mAb were added, and cultures were maintained for 5 days. Cell-free supernatants were analyzed for the secretion of innate cytokines by ELISA. **(A–C)** IL-6, IL-1β, and IL-23 production by monocytes and **(D–F)** IL-6, IL-1β, and IL-23 production by DCs (mean ± SEM, *n* = 7). **P* < 0.05; ***P* < 0.01; ****P* < 0.001; ns, not significant; as determined by one-way ANOVA.
